# Exogenous 24-Epibrassinolide Interacts with Light to Regulate Anthocyanin and Proanthocyanidin Biosynthesis in Cabernet Sauvignon (*Vitis vinifera* L.)

**DOI:** 10.3390/molecules23010093

**Published:** 2018-01-09

**Authors:** Yali Zhou, Chunlong Yuan, Shicheng Ruan, Zhenwen Zhang, Jiangfei Meng, Zhumei Xi

**Affiliations:** 1 College of Enology, Northwest A&F University, Yangling 712100, China; zhouyali@nwafu.edu.cn (Y.Z.); yuanchl69@nwsuaf.edu.cn (C.Y.); zhangzhw60@nwsuaf.edu.cn (Z.Z.); 2 Shaanxi Engineering Research Center for Viti-Viniculture, Yangling 712100, China; 3 Chateau Changyu Rena Co., Ltd., Xianyang 712000, China; ruanshicheng@sina.cn

**Keywords:** grape, anthocyanin biosynthesis, VvBZR1, VvHY5, 24-Epibrassinolide

## Abstract

Anthocyanins and proanthocyanidins (PAs) are crucial factors that affect the quality of grapes and the making of wine, which were stimulated by various stimuli and environment factors (sugar, hormones, light, and temperature). The aim of the study was to investigate the influence of exogenous 24-Epibrassinolide (EBR) and light on the mechanism of anthocyanins and PAs accumulation in grape berries. Grape clusters were sprayed with EBR (0.4 mg/L) under light and darkness conditions (EBR + L, EBR + D), or sprayed with deionized water under light and darkness conditions as controls (L, D), at the onset of veraison. A large amount of anthocyanins accumulated in the grape skins and was measured under EBR + L and L treatments, whereas EBR + D and D treatments severely suppressed anthocyanin accumulation. This indicated that EBR treatment could produce overlay effects under light, in comparison to that in dark. Real-time quantitative PCR analysis indicated that EBR application up-regulated the expression of genes (*VvCHI1*, *VvCHS2*, *VvCHS3*, *VvDFR*, *VvLDOX*, *VvMYBA1*) under light conditions. Under darkness conditions, only early biosynthetic genes of anthocyanin biosynthesis responded to EBR. Furthermore, we also analyzed the expression levels of the BR-regulated transcription factor VvBZR1 (Brassinazole-resistant 1) and light-regulated transcription factor VvHY5 (Elongated hypocotyl 5). Our results suggested that EBR and light had synergistic effects on the expression of genes in the anthocyanin biosynthesis pathway.

## 1. Introduction

**Figure 1:**
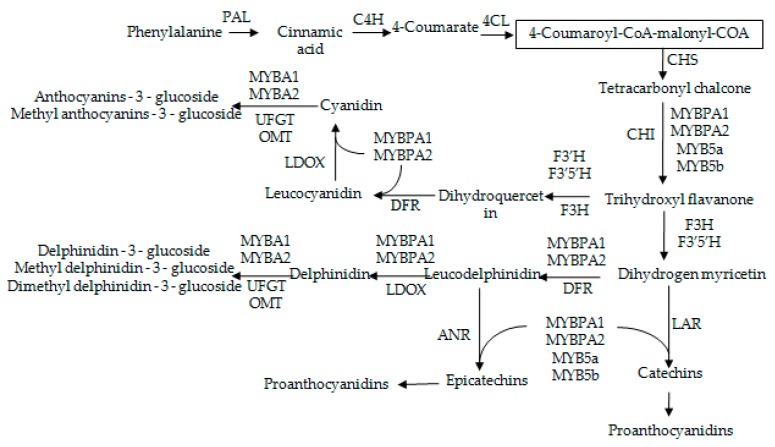
Biosynthetic pathway of anthocyanins and proanthocyanins in grape. Notes: Transcription regulators: VvMYBPA1, VvMYBPA2, VvMYBA1, VvMYBA2, VvMYB5a, VvMYB5b. PAL: phenylalanine ammonia-lyase, CHI: chalcone isomerase, DFR: dihydroflavonol 4-reductase, LAR: leucoanthocyanin reductase, ANR anthocyanin reductase, UFGT UDP-glucose: flavonoid 3-*O*-glucosyltransferase, LDOX: leucoanthocyandin dioxygenase.

Anthocyanins and proanthocyanidins (PAs) are crucial factors that affect the quality of grapes and the making of wine [1,2,3]. The accumulation of anthocyanin is stimulated by various stimuli: sugar, plant hormones and other environmental factors (light, UV irradiation, temperature, wounding, drought) [4,5,6,7,8,9,10,11]. To promote anthocyanin accumulation, application of plant growth regulators has been proposed as an economically viable alternative. The biosynthesis mechanisms of anthocyanins and PAs has been deeply studied [2,12,13,14,15,16]. The anthocyanins and PAs are synthesized via the phenylpropanoid pathway and flavonoids pathway in the cytoplasm as shown in Figure 1 [17]. The biosynthetic pathway leading to anthocyanins and PAs is well known and the key regulatory genes controlling the pathway have been reported [18,19]. In anthocyanin biosynthetic pathway, genes encoding enzymes are divided into two groups: the upstream genes of anthocyanin biosynthetic pathway, such as chalcone synthase (CHS) and chalcone isomerase (CHI), and the downstream genes of anthocyanin biosynthetic pathway including dihydroflavonol reductase (DFR), leucoanthocyandin dioxygenase (LDOX), and UDP-glucose: flavonoid-3-*O*-glucosyl transferase (UFGT) [15,20,21,22,23].

As reported previously, plant hormones including brassinosteroids (BRs), abscisic acid(ABA) and ethylene, etc., also regulate anthocyanin biosynthesis [24,25]. Brassinosteroids (BRs) has been considered the sixth steroidal hormone and has been intensively researched; it regulates a wide range of physiological processes and plays vital roles in plant growth and development [26,27,28,29,30]. In recent years, the effects of BRs on fruit growth and development have been investigated. BRs are used for the development and ripening of fleshy fruit, such as tomatoes [31], strawberries [32] and apples [33]. Exogenous application of BRs significantly promoted grapes ripening and enhanced the anthocyanin content [27,34]. BRs, through BR signaling mainly via BZR1 and BES1 (BRI1-EMS-suppressor 1), regulate a large number of genes involved in plant developmental and physiological processes. Thus, the transcription factors that directly regulate the expression of the structural genes in the development of plants have been identified in many species [35]. However, the molecular mechanism for the regulation of anthocyanin accumulation in grape by VvBZR1 remains unknown.

Another important environmental factor in anthocyanin synthesis is light. Understanding of the light-mediated mechanism involved in the regulation of anthocyanin biosynthesis in fruits has increased markedly [19,36]. Shin et al. (2007) reported that the combination of HY5 (Elongated hypocotyl 5) and PIF3 (Phytochrome-interacting factor 3) bound directly to the promoters of anthocyanin structural genes such as CHS, CHI, F3H, DFR and LDOX in Arabidopsis thaliana [5,37]. Wang et al. (2016) reported that HY5 directly binds the MYBL2 promoter and represses its expression to activate anthocyanin biosynthetic pathway in *Arabidopsis thaliana* [38].

Recent studies suggested that hormones participating in light transduction pathways control anthocyanin accumulation or, alternatively, that hormones and photoreceptors shared common molecular targets regulating the response [35]. Many studies had been performed to determine the impact of BRs on phenolic accumulation in grape berries. However, there are few reports concerning the interrelationship of EBR and light on anthocyanin accumulation in grape skin. Thus, it has not yet been elucidated how flavonoid biosynthesis pathway genes respond to various combinations of EBR and light.

In the experiment presented in this study, the effects of exogenous EBR and light treatment on the anthocyanins and PA content as well as mRNA expression levels of structural genes were measured in grape skins. It clarified the interrelationship of EBR and light effects on anthocyanin accumulation and the expression of flavonoid-related genes. The purpose of this study is to provide physiological support for researching the regulatory mechanism of VvBZR1 and VvHY5 involved in anthocyanin synthesis. These findings provide new information about the relationships between EBR, light and anthocyanin accumulation in grape berry skin.

## 2. Results

### 2.1. Physiochemical Parameters

**Figure 2:**
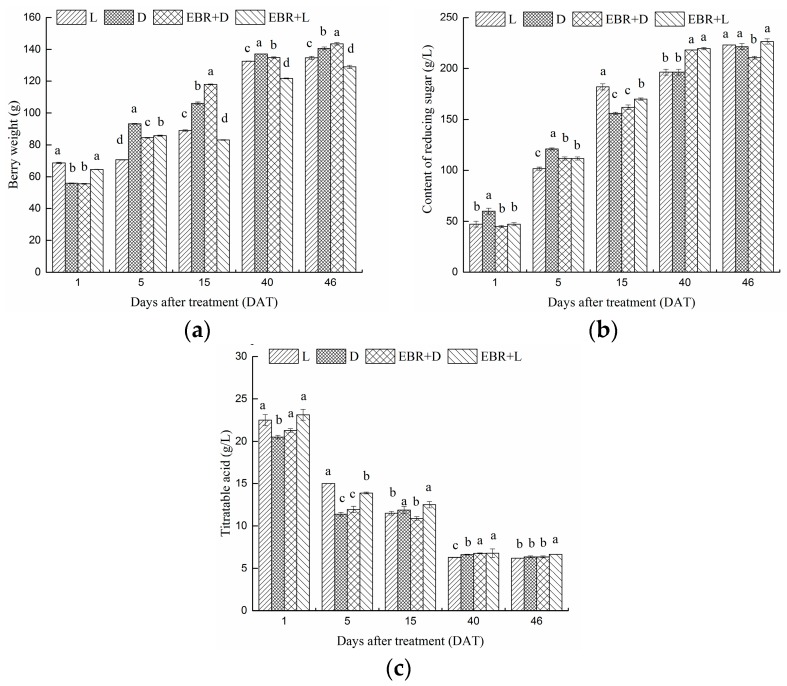
Effects of the four treatments on 100-berry weight (**a**); reducing sugar (**b**) and total acidity (**c**) in grape berry during fruit development. Data represent the mean of three replicates ± standard deviation (error bars). The different letters (a, b, c, d) indicate significant differences between treatments at *p* < 0.05 (Duncan’s multiple range test). DAT: Days after treatment. The same as belows.

Grapes were treated at E-L35 phenological stage and collected from 1 DAT to 46 DAT. EBR treatment significantly influenced 100-berry weight under the darkness condition (Figure 2). From fruit version to maturity, 100-berry weight was dramatically increased (46 DAT, E-L 38), then increased slightly and reached maximum value at harvest. Bagging without EBR (D) treatment had no significant effects on 100-berry weight, but EBR + D treatments increased the 100-berry weight at 15 and 46 DAT. At harvest (46 DAT, E-L 38), the mean 100-berry weight was 2.04% (EBR + D) higher than that of the bagging without EBR (D). The result suggests that EBR treatment could increase 100-berry weight under darkness condition. Correspondingly, the four treatments had the same effects on reducing levels of sugar and titratable acid, which were significantly increased under the light exposure with EBR (EBR + L) treatments relative to D, L (L: light exposure without EBR) and EBR + D (Figure 2b,c). The EBR treatments (EBR + D, EBR + L) enhanced reducing sugar accumulation and decreased the total acid content. During berry development, the reducing sugar content and total acid content in juice of EBR-treated (EBR + D, EBR + L) berries were significantly higher and lower, respectively, than that of the D and L treatments. In addition, light exposure significantly increased the content of reducing sugar during berry development (Figure 2). The result showed that EBR was more effective in the light than dark in the synthesis of the reducing sugar.

### 2.2. The Content of Anthocyanins and Proanthocyanidins in Grape Skins

**Figure 3:**
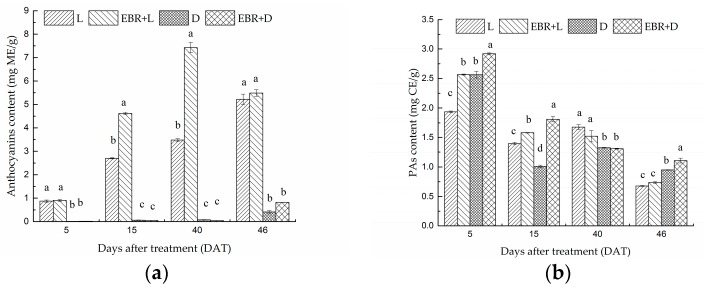
(**a**) Accumulation of anthocyanins per gram of skins dry weight in berry skins (cv. Cabernet Sauvignon) during development. The amounts are expressed as milligrams of cyanidin-3-monoglucoside equivalence (ME) per gram of dry berry skins (mg ME/g); (**b**) Accumulation of proanthocyanidins (PAs) per gram of skins dry weight in grape berries (cv. Cabernet Sauvignon) during development. The amounts are expressed as milligrams (+)-catechin equivalence (CE) per gram of dry berry skins (mg CE/g) (mean ± SE; *n* = 3). The different letters (a, b, c, d) indicate significant differences between treatments at *p* < 0.05 (Duncan’s multiple range test).

The effects of different treatments on anthocyanin content in grape skins at different ripening stages are shown in Figure 3a. Color development of berries started after 5 DAT and sufficient anthocyanins accumulation was observed, whereas D or EBR + D treatment suppressed anthocyanins accumulation severely (Figure 3a). The total anthocyanin content of berry skins were 0.0089, 0.699, 0.024 and 0.862 mg/g fresh weight in the 5 DAT in the D, L, EBR + D and EBR + L treatments, respectively (Figure 3a). The EBR treatment (EBR + D, EBR + L) significantly increased the content of anthocyanins in the skin, under light treatment, EBR treatment significantly increased the content of anthocyanins compared with the treatment of EBR + D. The total anthocyanin content in the EBR + L treatment was significantly higher than other treatments. The total anthocyanin content for each treatment in the 40 and 46 DAT was similar to that in the 5 and 15 DAT. Thus, the anthocyanin content was enhanced under the EBR + D treatment compared with D treatment at 46 DAT. These results suggested that light treatment with exogenous EBR treatment induced anthocyanin accumulation, but D treatment severely reduced it.

The effects of different treatments on PA content in grape skins at different ripening stages are shown in Figure 3b. The result suggested that EBR could increase the PA content significantly under darkness condition at 15 DAT and 46 DAT. Compared to D treatment, the PAs content in the skins was enhanced by 13.74%, 81.64%, 15.8% under the EBR + D treatment at 5 DAT, 15 DAT, 46 DAT, respectively.

### 2.3. Determination Monomer Anthocyanins Content in Grape Skins

**Figure 4:**
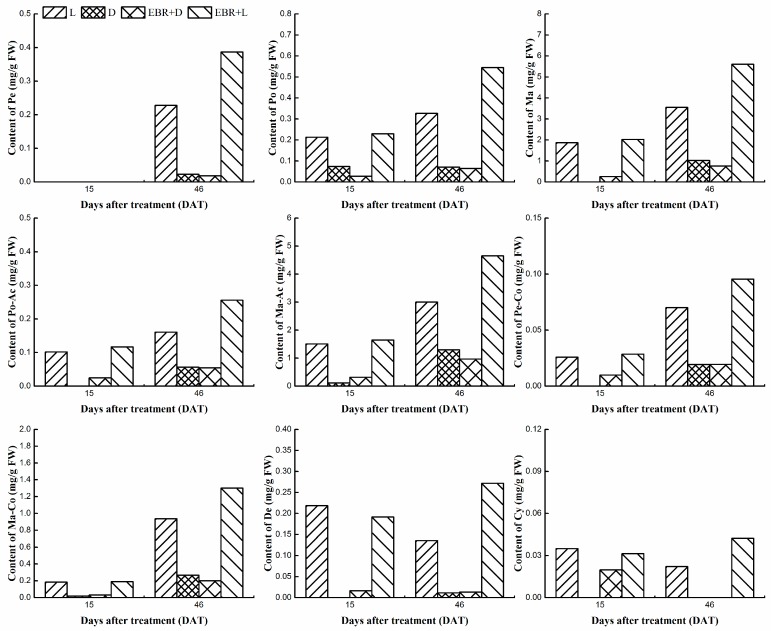
Individual anthocyanins content identified in Cabernet Sauvignon skins in 15 DAT and 46 DAT. Notes: Pe: 3’-Petunidin-3-*O*-glucoside; Po: Peonidin-3-*O*-glucoside; Ma: malvidin-3-*O*-glucoside; Po-Ac: Peonidin-3-*O*-(6-*O*-Acetyl)-glucoside; Ma-Ac: malvidin-3-*O*-(6-*O*-Acetyl)-glucoside; Pe-Co.: Petunidin-3-*O*-(6-*O*-Coumaryl)-glucoside; Ma-Co.: malvidin-3-*O*-(6-*O*-Coumaryl)-glucoside; De: Delphinidin-3-*O*-glucoside; Cy: Cyanidin-3-*O*-glucoside; D: dark; L: light; EBR + D: EBR + dark; EBR + L: EBR + light; Data represent the mean of three replicates ± standard deviation (error bars); FW: fresh weight.

Anthocyanins composition of grape skins was determined by LC-20AT HPLC system (Shimadzu, Kyoto, Japan). The anthocyanin compositions at 15 and 46 DAT upon different treatments are shown in Figure 4. The 15 DAT was the mid-maturity stage and the 46 DAT was the maturity stage, which were important stages in the development of grape berries. The results revealed that EBR treatment could significantly increase the content of monomer anthocyanins and the Ma-derivatives were the most abundant components among them (Figure 4). In L and EBR + L treatments, most of the Ma-derivatives were malvidin-3-*O*-glucoside and its acyl derivatives, the content was 3.55 mg/g fresh weight (FW), 3.85 mg/g FW at 15 DAT; 7.49 mg/g FW, 11.55 mg/g FW at 46 DAT, respectively. Thus, the fact was that malvidin-3-*O*-glucoside made up the vast majority of the total anthocyanins. The higher content of malvidin-3-*O*-glucoside and their acyl derivatives were detected in the skins of L and EBR + L treated berries, but the content of malvidin derivatives decreased in D and EBR + D treatments. Meanwhile, the result revealed that the content of Pn-derivatives, which is the second most abundant component in the skins, was 0.85 mg/g FW, 2.39 mg/g FW in 15 DAT, 46 DAT, respectively. However, both Cy-derivatives and De-derivatives were presented in low concentrations. In the skin of grape, malvidin-3-*O*-glucose was the major pigment, followed by malvidin-3-*O*-(6-*O*-acetyl)-glucoside (Figure 4). The results indicated that EBR and light firstly influenced malvidin-3-*O*-glucoside and its acyl derivatives accumulation and then promoted the biosynthesis of AT. It was apparent that the nine kinds of anthocyanidin derivatives were enhanced in response to EBR treatment (EBR + D, EBR + L). At harvest (46 DAT), the grape treated with L showed increases in total content of 1.91 and 3.64 times for Ma-derivatives and De-derivatives, respectively, in comparison with the D treatment. Likewise, the grape treated with EBR + L showed increases in total content of 5.01 and 7.25 times for Ma-derivatives and De-derivatives, respectively, in comparison with the EBR + D treatment, which demonstrated that EBR and light conditions affected the anthocyanins compositions in grape berries skins.

### 2.4. The Expression Patterns of Anthocyanins Biosynthetic Genes and Transcriptional Regulator Genes in Grape Skins

**Figure 5:**
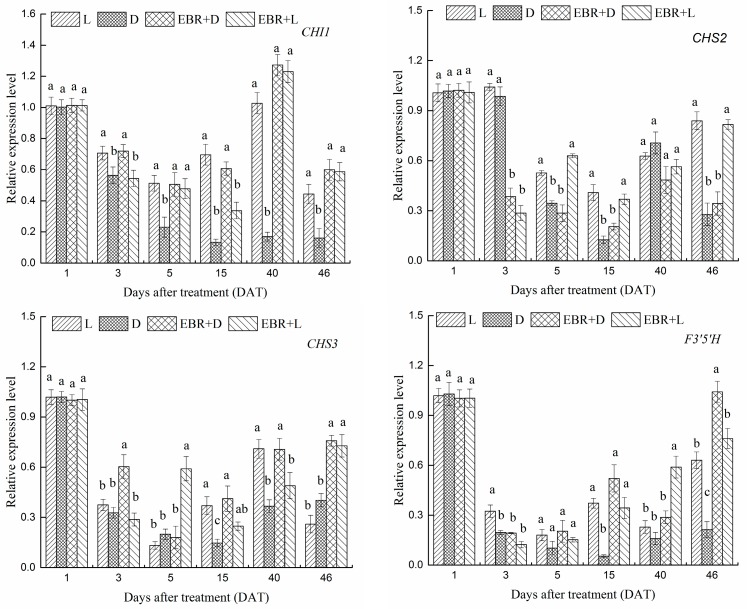

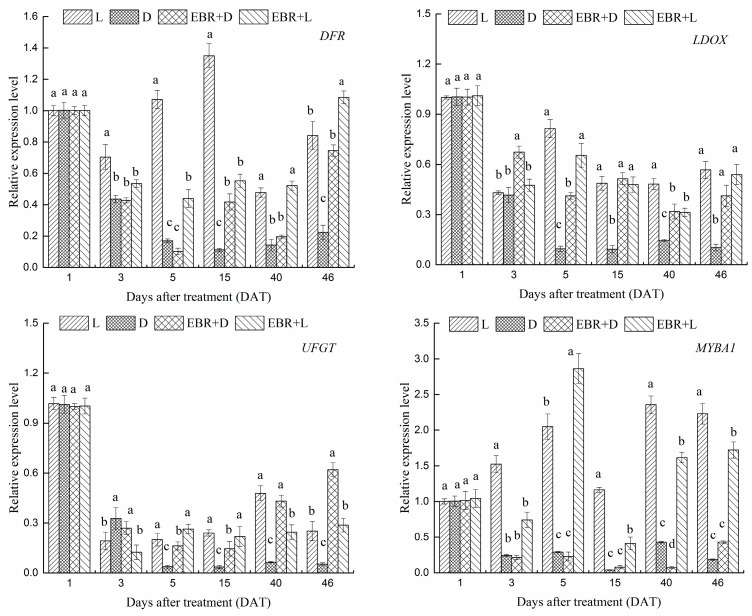
Transcript profiles of *VvCHI1*, *VvCHS2*, *VvCHS3*, *VvF3’5’H*, *VvDFR*, *VvLDOX*, *VvUFGT* and *VvMYBA1* as the molar ratio of the mRNA level to that of *VvGAPDH* in each sample (mean ± SE; *n* = 3). The different letters (a, b, c, d) indicate significant differences between treatments at *p* < 0.05 (Duncan’s multiple range test).

For further dissecting the EBR modulation of light controlling anthocyanin accumulation in grape, we measured the mRNA levels of *VvCHI1*, *VvCHS2*, *VvCHS3*, *VvF3’5’H*, *VvDFR*, *VvLDOX*, *VvUFGT* and *VvMYBA1* by real-time quantitative PCR (RT-qPCR) during berry development which are presented in Figure 5. The expression levels of anthocyanin biosynthesis structural genes were significantly higher under EBR treatment than under that D treatment, but having no clear effects compared to light treatment. Under different conditions of EBR treatments, the expression levels of *VvCHI1*, *VvCHS3*, *VvF3’5’H* and *VvUFGT* were not affected by light, while dark treatment significantly decreased the expression of *VvCHS2*, *VvDFR*, *VvLDOX* and *VvMYBA1*. The expression levels of *VvCHS2*, *VvDFR* and *VvLDOX* were the highest in EBR + L and drastically down-regulated by L or D treatment at 46 DAT. Meanwhile, L treatment improved the transcription levels of *VvCHI1*, *VvF3’5’H* and *VvDFR* by 1.75, 2, 2.82-fold than the D treatment respectively, and EBR + L treatment improved the transcription levels by 0.34, 0.21, 0.31-fold than L treatment respectively, at 46 DAT. For *VvCHI1*, *VvCHS3*, *VvF3’5’H* and *VvUFGT*, D treatment down-regulated gene expression, but the effects of D treatment was particularly dramatic under EBR treatment. The results indicated that both EBR and light are required to induce the expression of these genes. Although the expression levels of *VvCHS2* and *VvLDOX* were the highest in EBR + L, the effects of EBR and light treatment were unclear. Thus, many different expression patterns were observed among the flavonoid biosynthesis pathway genes. Meanwhile, under darkness conditions, several genes also exhibited up-regulated expression after EBR treatment. These genes also included *VvCHI*, *VvCHS3*, *VvF3’5’H*, *VvDFR*, *VvLDOX* and *VvUFGT*. Under darkness conditions, genes of the late-steps of anthocyanins biosynthesis, which played a more direct role in the biosynthesis of anthocyanins, showed significant change in expression with EBR treatment at 46 DAT.

As shown in Figure 5, the treatment of EBR + L also increased the mRNA expression level of the transcriptional regulator gene *VvMYBA1* at each sampling date, but D and EBR + D treatment severely suppressed the expression of this gene. The pattern of *VvMYBA1* expression across the four treatments was similar to that of the anthocyanins content and suggested that both EBR and light were needed to induce its expression. The expression level of *VvMYBA1* was significantly induced by light treatment, indicating that *VvMYBA1* might be a light-responsive gene.

### 2.5. The Expression Patterns of Pas Biosynthetic Genes and Transcriptional Regulator Genes in Grape Skins

**Figure 6:**
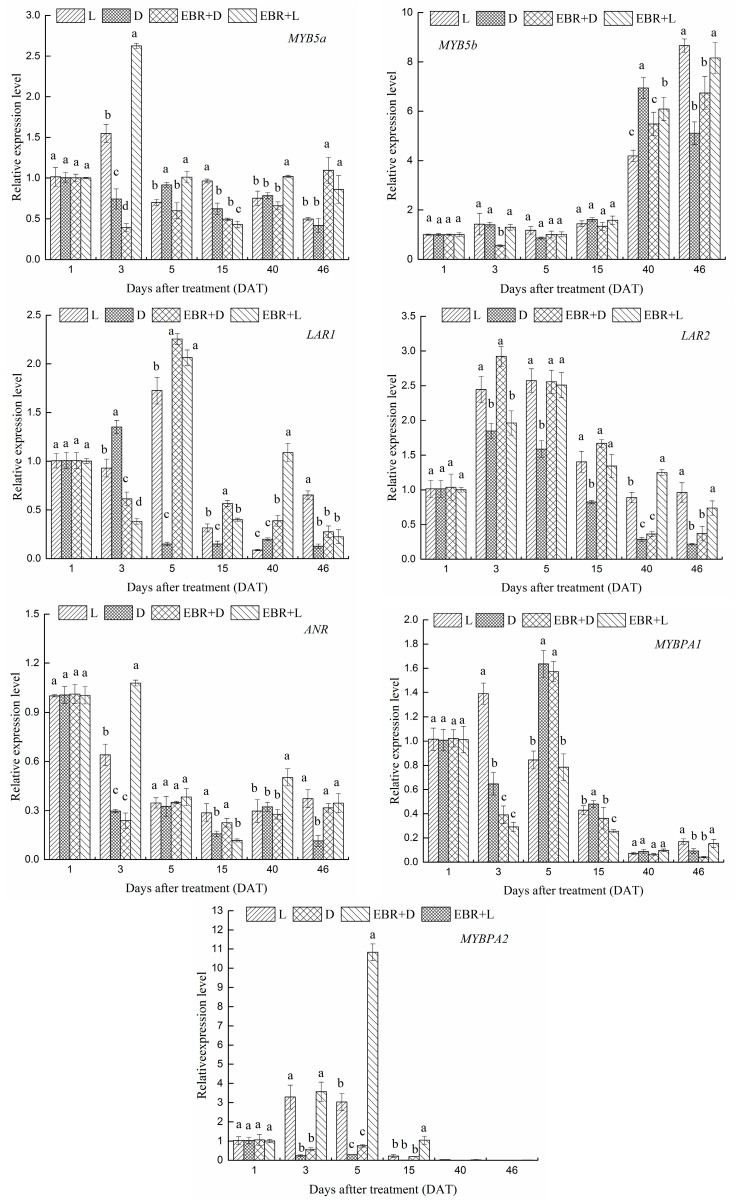
Transcript profiles of *VvMYB5a*, *VvMYB5b*, *VvLAR1*, *VvLAR2*, *VvANR*, *VvMYBPA1* and *VvMYBPA2* as the molar ratio of the mRNA level to that of *VvGAPDH* in each sample (mean ± SE; *n* = 3). The different letters (a, b, c, d) indicate significant differences between treatments at *p* < 0.05 (Duncan’s multiple range test).

We investigated the effects of the four treatments on the expression of PA biosynthesis genes (*VvLAR1*, *VvLAR2*, *VvANR*) and their transcriptional regulators (VvMYB5a, VvMYB5b, VvMYBPA1 and VvMYBPA2) by RT-qPCR in berry skins (Figure 6). The EBR + L treatment induced the expression of *VvMYB5a* and *VvMYB5b*, but D or L treatment severely suppressed the expression of these genes. The pattern of *VvMYB5a* and *VvMYB5b* across the four treatments was also similar to that of the anthocyanin content and suggested that both EBR and L were needed to induce its expression. Expression levels of VvMYBPA1 increased to a maximum at 5 DAT, and then declined to a low level after 15 DAT (Figure 6). As for VvMYBPA1, EBR and light treatments could not significantly induce its expression, in contrast, D treatment induced VvMYBPA1 synthesis from five to 15 DAT. At the beginning of veraison, EBR and light treatments could significantly induce the expression of VvMYBPA2 from three to 15 DAT, and the expression level of VvMYBPA1 nearly declined to zero after 40 DAT. The expression level of *VvANR* was higher in the EBR + D treatment than the D treatment at 46 DAT. Although the PA accumulation in the EBR + D treatment was severely suppressed (Figure 3b), the expression level of *VvANR* in the EBR + D treatment was only moderately suppressed. At 5 DAT, EBR treatments (EBR + D, EBR + L) significantly increased the expression levels of *VvLAR1* and *VvLAR2*. Thus, each of the PAs biosynthesis genes (*VvLAR1*, *VvLAR2*, *VvANR*) and their transcriptional regulators (VvMYB5a, VvMYB5b, VvMYBPA1 and VvMYBPA2) reacted differently to the light and EBR treatments.

### 2.6. Expression Patterns of VvBZR1, VvHY5 and VvBRI1 Transcriptional Regulator Genes in Grape Skins

**Figure 7:**
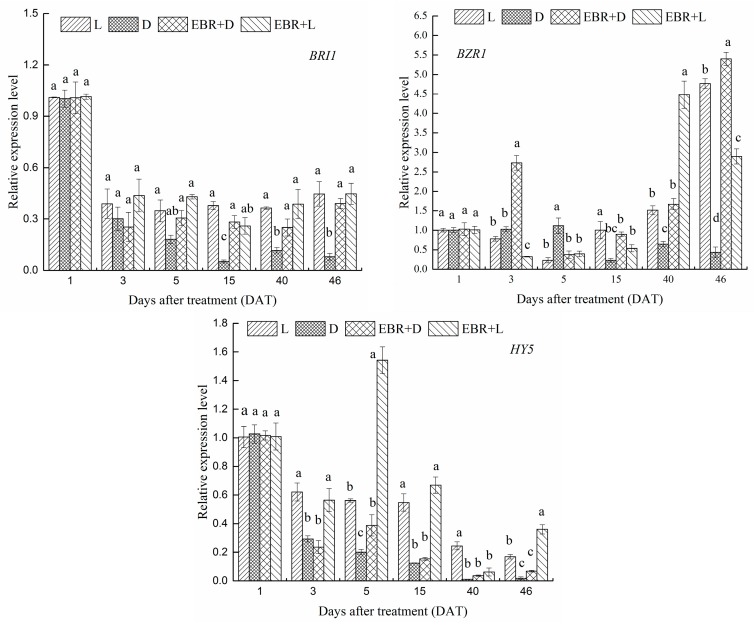
Transcript profiles of *VvBRI1*, *VvBZR1* and *VvHY5* as the molar ratio of the mRNA level to that of *VvGAPDH* in each sample (mean ± SE; *n* = 3). The different letters (a, b, c, d) indicate significant differences between treatments at *p* < 0.05 (Duncan’s multiple range test).

The effects of different treatments on the mRNA levels of *VvBRI1* (brassinosteroid insensitive 1), *VvBZR1* (brassinazole-resistant 1) and *VvHY5* (elongated hypocotyls 5) genes were determined by RT-qPCR during berry development. The expression level of *VvBZR1* during berry development is presented in Figure 7. As illustrated in Figure 7, the expression level of *VvBZR1* was at a relatively high level enhanced by EBR treatment at 40 and 46 DAT. However, the expression level of *VvBZR1* was at a relatively low level during veraison. Meanwhile, the result displayed that EBR + L treatment significantly increased the mRNA level of *VvHY5* at 5 DAT.

*VvBRI1* (brassinosteroid insensitive 1) is a LRR receptor kinase of BR receptor, which exists on the surface of the cell. Usually higher expression of upstream genes leads to higher expression of downstream genes. However, EBR and L treatments (EBR + D, EBR + L, L) have no significant effects on the mRNA levels of *VvBRI1* during berry development as can be observed in Figure 7, but compared to D treatment, EBR treatments (EBR + D, EBR + L) had significant effects on the mRNA levels of *VvBRI1*. This indicated that expression of *VvBRI1* might be up-regulated by light, independent of the treatment of EBR.

### 2.7. Hierarchical Clustering in the Profiles of All Genes Analyzed

**Figure 8:**
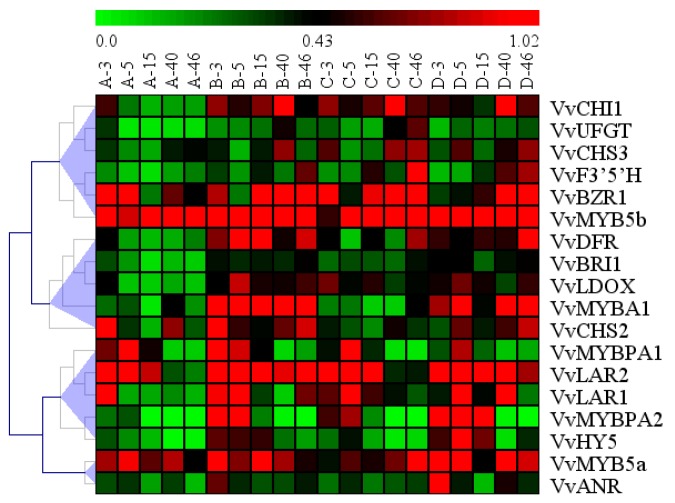
Hierarchical clustering of the transcript profiles of all genes. The relative expression levels of the genes after the four treatments compared to the reference gene, used in the original rank-based algorithm, were used for the hierarchical cluster analysis with Genesis. Red colors represent relatively higher transcript abundances, and green colors represent relatively lower transcript abundances. Sampling times and treatments are indicated at the top; the numbers A, B, C and D represent treatments D, L, EBR + D, EBR + L, respectively, and the numbers 3, 5, 15, 40 and 46 are days after treatment.

Hierarchical clustering was performed by Multiple Array Viewer to access the similarity of the profiles of gene expression in the four treatments. We identified four clusters (Figure 8). VvBZR1, the key transcription factor in BR signal transcription pathway was clustered with *VvMYB5b*, *VvF3’5’H*, *VvCHS3*, *VvUFGT* and *VvCHI1*. The expression level of *VvMYB5b* was similar to *VvBZR1*, which indicated that *VvMYB5b* had a relation with *VvBZR1*. Furthermore, we could infer that PA synthesis hada relationship with *VvBZR1* at the transcriptional level. The typical anthocyanin structure genes (*VvCHS2*, *VvMYBA1*, *VvDFR*, *VvLDOX*) were clustered in a group with *VvBRI1*. The genes in this group were strongly induced by EBR and light treatment (EBR + D, EBR + L), indicating that the EBR treatment enhanced their expression and signal transduction pathway which is involved in the anthocyanin accumulation and PAs biosynthesis. The PAs biosynthesis genes (*VvLAR1*, *VvLAR2*) and their transcriptional regulators (VvMYBPA1 and VvMYBPA2) were clustered in a group with *VvHY5*. The expression pattern of genes was similar to that of the PAs content in this group. Additional PAs structure genes *VvANR* and *VvMYB5a* were clustered into another group. The genes were up-regulated by EBR + D treatment than EBR + L treatment but were down-regulated under D treatment at 46 DAT, further supporting that EBR was the main stimulus in the biosynthesis of PAs.

## 3. Discussion

In this study, the content of PAs, anthocyanins and monomer anthocyanins were determined. EBR treatment improved accumulation of phenolic compound in the skins of the grape berries not only when spraying at maturity [27] but also at the stage of veraison. Under light exposure, EBR treatment markedly increased the accumulation of PAs, anthocyanins and monomer anthocyanins. These results, which were in agreement with previous studies, showed that artificial shading of fruits would decrease the content of phenolic compound, and light exposure would increase phenolic compound content [7,39,40,41]. Before maturity, EBR + L treatment enhanced accumulation of phenolic compound (PAs, anthocyanins) in the skins. At harvest, the content of PAs, anthocyanins and monomer anthocyanins were slightly decreased; this is due to the polyphenols in skins being transferred to the seeds during the growing process, which was corroborated by previous studies [42,43,44].

It could be observed from Figure 5 that light and EBR were essential in anthocyanin biosynthesis. EBR treatment (EBR + D, EBR + L) was involved in the regulation of *VvCHI1*, *VvCHS3*, *VvF3’5’H*, *VvDFR* and *VvUFGT* expression and reached a peak at the veraison; thereafter, the mRNA levels decreased rapidly then constantly increased until harvest (46 DAT, E-L38) compared with those of the D treatment. In this study, the expression levels of *VvCHI1*, *VvCHS2*, *VvCHS3*, *VvDFR* and *VvLDOX* were up-regulated by light and EBR treatments. However, the expression levels of *VvCHI1*, *VvCHS3*, *VvF3’5’H* and *VvUFGT* were up-regulated by EBR + D higher than EBR + L, which was similar to *VvBRI1* (Figure 8).

We investigated the effects of different treatments on the expression of PAs biosynthetic genes (*VvLAR1*, *VvLAR2*, *VvANR*) and their transcriptional regulators (VvMYB5a, VvMYB5b, VvMYBPA1 and VvMYBPA2). The expression levels of *VvLAR1*, *VvLAR2* were higher in early development berries (Figure 6) than harvest (E-L38), which was supported by previous research [15,34,45,46]. The research showed that *VvLAR2* mRNA level was always higher than that of *VvLAR1* under the same treatment at harvest, thus, light exposure could increase the *VvLAR2* mRNA level, however, EBR + D treatment significantly increased the *VvLAR1* mRNA level at 5, 15 DAT. Our results predicted that the *VvLAR2* might play more important roles in two LAR isoforms involved in the regulation of the PAs biosynthesis pathway [46,47]. In the PAs biosynthesis pathway, ANR is the other crucial catalyzing enzyme. During berry development, the *VvANR* mRNA level was increased markedly in the EBR treatments (EBR + D, EBR + L), which contributed to the higher levels of *VvLAR1* and *VvLAR2* mRNA under different conditions. These effects of EBR were similar to those of abscisic acid (ABA) and methyl jasmonate (JA) on the synthetic process of proanthocyanidins and anthocyanins [48,49,50]. In grapes, VvMYB5a and VvMYB5b were transcriptional regulators related to PA metabolism [15,51], and previous studies have already identified VvMYBPA1 and VvMYBPA2 as the transcription factors involved in the regulation of the proanthocyanidin pathway during grape development [52,53]. Bogs et al. found that the expression level of VvMYBPA1 in skins was relatively low before veraison, increased to a maximum two weeks after veraison, and then declined to a low level. Terrier et al. found that VvMYBPA2 expression mostly existed in the skin of very young berries, and the expression level declined to very low levels after veraison. The treatments were carried out at the beginning of veraison, so the expression levels of VvMYBPA1 and VvMYBPA2 were consistent with that of Bogs et al. and Terrier et al., respectively [52,53]. As for VvMYBPA1, D treatment induced its synthesis from five to 15 DAT (Figure 6), maybe due to the maturity processing of grape being delayed by darkness treatment. It is worth noting that the expression of VvMYB5a and VvMYB5b activated *VvLAR*, *VvANR* and *VvCHI1* [1,15,17,54]. Thus, as illustrated by Figure 8, the transcription profiles of transcriptional regulator VvMYB5b was consistent with VvBZR1. Deluc et al. (2008) reported that the transcription factor VvMYB5b contributed to the regulation of anthocyanins and proanthocyanidins biosynthesis in development grape berries [15]. We inferred that VvBZR1 had a relationship with VvMYB5b in regulation of biosynthesis of anthocyanins and proanthocyanidins. VvBZR1 is a key transcription factor associated with the BR signal transduction pathway, which plays an important role in BR-regulated genes [55,56]. The results showed that different treatments made the genes expression different up to 46 DAT.

It is known that the effect of the EBR treatment on the expression of genes and transcription factors is almost nonexistent after 48 hr or even 24 hr treated. In this study, both of the PA biosynthesis genes (*VvLAR1*, *VvLAR2*, *VvANR*) and their transcriptional regulators (VvMYB5a, VvMYB5b, VvMYBPA1 and VvMYBPA2) showed none significant difference at 1 DAT. At 3 DAT, EBR + L treatment significantly increased the expression levels of *VvLAR1* and *VvANR* compared to L treatment. Furthermore, at 5 DAT, EBR + L treatment significantly up-regulated the expression levels of *VvLAR1*, *VvCHS3*, *VvUFGT*, VvMYBA1, VvMYB5a and VvMYBPA2 compared to L treatment. These results indicated that EBR could promote the expression of genes which control anthocyanin and PAs synthesis in the early days after ERB treatment. The differences in the expression of genes and transcription factors in our research up to 46 DAT may be because exogenous EBR promoted the synthesis of endogenous BRs or other endogenous hormones in the grape in later period.

Currently, the molecular mechanism of EBR enhancing the anthocyanin content in the skins is not well understood. We investigated the effects of EBR on the expression of *VvBZR1*, *VvHY5* and *VvBRI1* genes. *VvHY5* was extensively researched as a transcription factor involved in promoting photomorphogenesis. As shown in Figure 5 and Figure 8, light exposure can significantly influence the expression level of *VvHY5* and enhance the content of anthocyanins in grape berries, and photomorphogenic factors, HY5 and PIF3 bind directly to the promoters of anthocyanins structural genes such as *CHS*, *CHI*, *F3H*, *DFR* and *LDOX* in *Arabidopsis thaliana* [37]. Meanwhile, previous research reported that *VvHY5* was involved in flavonoid biosynthesis depending on light [5,57]. Thus, the results shown in Figure 4, Figure 5 and Figure 7, suggested that EBR treatment could increase the content of anthocyanins, up-regulate the expression of *VvHY5* and anthocyanin structural genes. On the basis of these finding, we inferred that *VvBZR1* could enhance the expression of *VvHY5*, which presented opposite trends with *VvBZR1*, further up-regulate the expression of anthocyanins structural genes and increase the content of anthocyanins either at transcription level or protein level.

In the study, we examined whether light participated in the process of BR signal transduction. EBR + L treatment significantly increased the mRNA expression levels of *VvBRI1* and *VvBZR1*, suggesting that light did significantly affect BR signal transduction upstream of *VvBZR1* at harvest and significantly increase the content of anthocyanins. In addition, EBR and light affected the expression level of *VvHY5*. These results supported the interaction between BR signaling, light signaling and anthocyanin biosynthesis pathways. However, how exactly VvBZR1 functions during anthocyanins biosynthesis process is still an open question. Future efforts will be focused on the mechanism by VvBZR1 and VvHY5 regulating anthocyanin biosynthetic pathway to illustrate how BR and light signaling pathways interplay to control the accumulation of anthocyanins.

## 4. Materials and Methods

### 4.1. Sample Treatments

Cabernet Sauvignon (*Vitis vinifera* L.) berries were sampled from a commercial vineyard in Xianyang, Shaanxi Province, China (34°650′ N, 108°750′ E). The grapevines were planted in 2009 (Seven-year-old own-rooted grapevines) and employed the single cordon pruning method. The grapevines were planted in North-South oriented rows with spacing of 1.0 m within rows and 2.5 m between rows. The grapevines were trained on a vertical shoot-positioning system with a pair of wires. Three blocks were chosen as biological replicates from one field randomly, and 30 plants of each block received a different spray treatment: light exposure with EBR and without EBR, bagging with and without EBR, once 0.4 mg/L 24-Epibrassinolide (Ruibio, Cologne, Germany) were applied at the onset of veraison. Shading material was 20 cm × 30 cm fruit bag with black double-layer inside.

### 4.2. Experimental Design and Samples Collection

Stock solutions of EBR were prepared by dissolving EBR in 1 mL of 98% ethanol. The control stock solution contained 1 mL of 98% ethanol without addition of EBR. Each stock solution was mixed with 1 mL of Tween 80 and diluted to 1 L with sterilized deionized water. In this study, the treatments were carried out at the beginning of veraison (softening of 10% of the berries), each solution was sprayed to cover the entire surface area of the berries in the cluster, after the surface was dry, bagging was carried out. 24-Epibrassinolide affects the accumulation of regulatory-gene transcripts at the beginning of veraison [34]. The application dates were 27 July in 2016. All spray applications were carried out at sunset.

Grapes were collected at E-L35 phenological stages (1, 3, 5 days after treatment-DAT), E-L36 (15 DAT), E-L37 (40 DAT) and E-L38 (46 DAT) [58]. Each samples consisted of 300 berries randomly from inside and outside of the cluster, the top, the bottom, and the middle of the cluster. These samples were stored at −20 °C for analysis of the phenolic compounds. In addition, another 60 berries in each treatment were divided into three groups as three replicates, then, the samples were frozen in liquid nitrogen and stored at −80 °C for RNA extraction and quantification of gene expression by real-time quantitative PCR.

### 4.3. Determination the Physicochemical Indices of Berries

The 100-berry weight for each replicates per treatment was recorded after blotting of residual moisture on the skins surface. Berry juice was collected and used to assay the content of reducing sugars and titratable acids, which were analyzed in accordance with the methods proposed by OIV (2012).

### 4.4. Extraction of Phenolic Compounds From Grape Skins

Phenolic compounds of grape in skins were extracted according to the methods proposed by Di Stefano and Cravero [59] with minor modifications. Grape skins and seeds of about 90 berries were carefully removed using razor blades. Then the residual water on the surface of grapes was dried and weighed dried skins. Added to 30 mL buffer solution (12% *v*/*v* ethanol + 600 mg/L Sodium metabisulfite + 5 g/L Tartaric Acid, 1 M NaOH adjust pH to 3.20), and put in swing bed (100 r/min, 25 °C), extracted for three days, collected the supernatant, finally, placed in −20 °C stored keep away from light before use.

### 4.5. Determination of Phenolics Content

Total proanthocyanidins content (PAs) were performed as described previously [60,61] with minor modifications. Buffer A was washing buffer containing 200 mM acetic acid and 170 mM sodium chloride, pH adjusted to 4.9 with sodium hydroxide. Buffer B was a model wine (5.0 g/L potassium bitartrate and 12% (*v*/*v*) ethanol, pH was adjusted to 3.3 with HCl. Buffer C was a suspending buffer consisting 5% (*v*/*v*) triethanolamine and of 5% (*w*/*v*) sodium dodecyl sulphate, pH was adjusted to 9.4 with HCl. Ferric chloride reagent was made by 0.01 M HCl and 10 mM ferric chloride.

For PAs determination, a protein solution for tannin precipitation was prepared by dissolving Bovine serum albumin (BSA) in a buffer A, giving a final protein concentration of 1.0 mg/mL. The skins extract was added 200 μL extract sample and 4.0 mL of the protein solution in a 1.5 mL microfuge tube. After incubating for 15 min with slow agitation at room temperature, the mixture was centrifuged at 14,000× *g* for 5 min at 4 °C. After the precipitate was washed with buffer A three times and then resolubilization in 3.5 mL of buffer C. The absorbance of the solution was read at 510 nm for as tannin background A510. Then, 0.25 mL of ferric chloride reagent was added and shaken for 10 min in the dark. The absorbance of the solution were read at 510 nm as tannin final A510. Buffer C was used as a blank and read at 510 nm for tannin initial A510. After the incubation period the absorbance at 510 nm was determined in Shimadzu 640 spectrophotometer. PAs values are reported in catechin equivalents (C.E.) as described here.

The absorbance for PAs = [(tannin final A510) − (tannin initial A510)] − (tannin background A510) × 0.875.

Total anthocyanins content was estimated using the pH differential method [62] with minor modification. Each grape extract was diluted 20-times with buffers at pH 1.0 and 4.5. The absorbance was measured at 520 and 700 nm in both pH 1.0 and 4.5 buffers. The anthocyanins (expressed in terms of cyanidin-3-glucoside) was calculated using the following formula: A = (A520 − A700) pH 1.0 −(A520 − A700) pH 4.5, the anthocyanins content was expressed as Milligrams of malvidin-3-monoglucoside equivalence per berry (mg ME/berry) and calculated using the equation anthocyanins content = A × DF × MW × 1000/(ε × C), Where: MW is the molecular weight of cyanidin-3-glucoside (449 g/mol), DF is the dilution factor, ε is the molar extinction coefficient of cyanidin-3-*O*-glucoside (29,600) and C is the concentration of extracted volume.

### 4.6. Determination the Content of Monomer Anthocyanins

The chromatographic analyses of anthocyanins were performed using LC-20AT HPLC system (Shimadzu, Japan), consisting of Pump LC-20AT, Central controller CBM-20A, Auto sampler SIL-20A, Column oven CTO-20A, Detector (PDA) SPD-M20A, equipped with a reversed phase column (Synergi Hydro-RP C18, 250 × 4.6 mm, 4 μm). The mobile phase was ultrapure water:acetonitrile:methanoic acid (800:100:25) as solvent A, and ultrapure water:acetonitrile:methanoic acid (400:500:25) as solvent B. The elution profile had the following proportions (*v*/*v*) of solvent B: 0.00–15 min, 0%–10%; 15–30 min, 10%–20%; 30–45 min, 20–35%; 45–46.00 min, 35%–100%; 46.00–50.00 min, 100%. The column was held at 35 °C and at a flow rate of 1 mL/min. The injection volume was 20 µL and analyses were detected at 520 nm. Before injection, the extracts were filtered through 0.22 m filters (cellulose acetate and nitrocellulose, CAN).

All phenolic compounds were identified by comparison of their order of elution and retention time with those of standards, the weight of molecular ion and the fragment ion with standards and references. Quantitative determinations were made by the external standard method with the commercial standards. The calibration curves were obtained by injecting of standard solutions under the same conditions as for the samples analyzed, observing the range of concentrations. Anthocyanins, flavan-3-ols, were respectively expressed as micrograms of malvidin-3-*O*-glucoside (ME), catechin equivalence (CE)/L of grape skins.

### 4.7. RNA Extraction and cDNA Synthesis Real-Time Quantitative PCR Analysis

Expression levels of the anthocyanins and PA biosynthesis genes *VvCHI1*, *VvCHS2*, *VvCHS3*, *VvF3’5’H*, *VvDFR*, *VvLDOX*, *VvUFGT*, *VvMYBA1*, *VvLAR1*, *VvLAR2*, *VvANR* and the transcription factor genes VvMYB5a, VvMYB5b in grape skins were measured by real-time quantitative PCR (RT-qPCR), using the IQ-SYBR Green Supermix on a MyIQTM Single Colour IQ5 Real-Time PCR Detection System (Bio-Rad Laboratories, Berkeley, CA, USA) monitored via the IQ5 Standard Edition Optical System Software 2.0 (Bio-Rad). The primers (Supplementary Materials Table S1) designed by Primer Premier 5. The two-step RT-qPCR Reagent Kit (Vazyme Biotech Co., Ltd., Nanjing, China) was used in accordance with the manufacturer’s instructions. The reaction mixture (20 μL) contained 2 μL cDNA (20 times dilution), 0.8 μL of each primer suspension (10 μmol/L) (Supplementary Materials Table S2), 10 μL 2 × Premix (Vazyme), and 7.2 μL ddH_2_O. The reaction conditions were as follows: 95 °C for 30 s, followed by 40 cycles of 95 °C for 10 s, 58 °C for 30 s. A melting cycle from 60 to 95 °C as the last step was used to check the specificity of each gene product, assisting by gel electrophoresis and sequence analysis. The annealing temperature (58 °C) was determined when designing the primers and by preliminary experiments. Expression levels for each gene were normalized to constitutively expressed transcripts by VvGAPDH, and calculated using the equation $2^{-∆∆Ct}$, in which ∆∆Ct = (CT, Target CT, VvGAPDH) Time X− (CT, Target CT, VvGAPDH) Time 0 [63]. Time X is any time point, and Time 0 represents E-L35. Three PCR replicates were conducted per sample and the fold change in each target gene of time 0 was set to 1. For each experiment, data were analyzed separately.

### 4.8. Statistical Analysis

Data were analyzed using SPSS 19.0 software (SPSS, Chicago, IL, USA). The significance of the difference between each treatment was determined by one-way analysis of variance (ANOVA) and Duncan’s new multiple range tests at the 0.05 and 0.01 significance levels. The data for physiochemical parameters, AT and PA content, the gene-expression profiles and monomer anthocyanins were analyzed using OriginPro2016. Data were expressed as the mean values of triplicate experiments and different letters (a, b, c, d) indicate a significant difference between treatments and the control.

## 5. Conclusions

In these experiments, the effects of exogenous EBR and light treatments on anthocyanin and PAs accumulation and the transcript patterns of relative structural genes were studied. The results indicated that EBR interacts with light to induce the synthesis of anthocyanins and PAs by up-regulating biosynthesis genes, which promoted the accumulation of anthocyanins, PAs and monomer anthocyanins in grape skins. In addition, we also found that both EBR and light affected the expression levels of VvBZR1 and VvHY5, which it may help us to illustrate that BR and light signaling pathways interplay to control the accumulation of anthocyanins in our future studies.

## Supplementary Materials

The supplementary materials are available online.
