# Supplementary material for: Exogenous 24-Epibrassinolide Interacts with Light to Regulate Anthocyanin and Proanthocyanidin Biosynthesis in Cabernet Sauvignon (Vitis vinifera L.)
Source: Molecules. 2018 Jan 9;23(1):93. doi: 10.3390/molecules23010093 (PMC6017727; doi:10.3390/molecules23010093)
Supplement: Supplementary file 1 [file molecules-23-00093-s001.pdf]

Table 1 the primer sequence

| Gene ID | Gene name | Primer | Sequence | |
|---|---|---|---|---|
| *CB975975* | *VvBRI 1* | F | CCTCCAAGCCTCATAACTGC | This study |
| | | R | CGAGCATTGCAGAGGTCATC | |
| XM_003632950 | *VvBZR1* | F | AGAGAAGGTGCGAAGCGTGAGT | This study |
| | | R | GAAGCGAAACCAGAAGAGGGAG | |
| *XM_002273693* | *VvPIF3* | F | CCAATACAACTAACAACGGC' | This study |
| | | R | ACATACAGAGGCAGACAATAAT | |
| ***XM_002275530*** | *VvHY5* | F | TTCTCACCCTTGTTTGGTTGCC | This study |
| | | R | TCTCCTCGTCACTCTCCATTCC | |
| AB066275 | *VvCHS2* | F | GAAGATGGGAATGGCTGCTG | Jeong et al. 2004 |
| | | R | AAGGCACAGGGACACAAAAAG | |
| AB066274 | *VvCHS3* | F | GGGCAGGCACTTTTTGGC | This study |
| | | R | TCGCTGTCGGGGAGGATT | |
| AB097923 | *VvMYBA1* | F | TAGTCACCACTTCAAAAAGG | Jeong et al. 2004 |
| | | R | GAATGTGTTTGGGGTTTATC | |
| *AY555190* | *VvMYB5a* | F | GTGCAGCAGCCATCTAATGTG | Matus et al. 2008 |
| | | R | GCAGCAGGTTCCCAGACAGT | |
| *AY899404* | *VvMYB5b* | F | GGTGTTCTTTAATTTGGCTTCA | Deluc et al. 2008 |
| | | R | CACAACAACACAACCACATACA | |
| X75963 | *VvCHI1* | F | GCTCCACCAACGACCTCTTCCT | This study |
| | | R | CCTCCACAGTCTTGCCCTTCCA | |
| ***DQ298201*** | *VvF3´5´H* | F | GAAGTTCGACTGGTTATTAACAAAGAT | Sinilal et al. 2011 |
| | | R | AGGAGGAGTGCTTTAATGTTGGTA | |
| X75964 | *VvDFR* | F | GAAACCTGTAGATGGCAGGA | Jeong et al. 2004 |
| | | R | GGCCAAATCAAACTACCAGA | |
| X75966 | *VvLDOX* | F | AGGGAAGGGAAAACAAGTAG | Jeong et al. 2004 |
| | | R | ACTCTTTGGGGATTGACTGG | |
| AF000372 | *VvUFGT* | F | GGGATGGTGATGGCTGTGG | Jeong et al. 2004 |
| | | R | ACATGGGTGGAGAGTGAGTT | |
| AJ865336 | *VvLAR1* | F | CAGGAGGCTATGGAGAAGATA | Zhang et al. 2013 |
| | | R | ACGCTTCTCTCTGTACATGTTG | |
| AJ865334 | *VvLAR2* | F | TCTCGACATAAATGATGATGTG | Zhang et al. 2013 |
| | | R | TGCAGTTTCTTTGATTGAGTTC | |
| AB199315 | *VvANR* | F | CAATACCAGTGTTCCTGAGC | Zhang et al. 2013 |
| | | R | AAACTGAACCCCTCTTTCAC | |
| | *Vv*GAPDH(m) | F | TTCTCGTTGAGGGCTATTCCA | EC958777 EC931777 |
| | | R | CCACAGACTTCATCGGTGACA | EC925368 CB973647 |

Table 2 the reaction mixture

| | |
|---|---|
| 2×ChamQ SYBR qPCR Master Mix | 10.0 μL |
| Primer1 (10 μM) | 0.4 μL |
| Primer2 (μM) | 0.4 μL |
| Template DNA/cDNA | 2 μL |
| $ddH_2O$ | 7.2 μL |
